# Exploring the mechanisms and targets of proton pump inhibitors-induced osteoporosis through network toxicology, molecular docking, and molecular dynamics simulations

**DOI:** 10.3389/fphar.2025.1592048

**Published:** 2025-05-12

**Authors:** Yaqi Mu, Yaqi Zhou, Xinan Zhang, Yiming Shao

**Affiliations:** ^1^ Department of Pharmacy, Zhengzhou Central Hospital Affiliated to Zhengzhou University, Zhengzhou, China; ^2^ Department of Orthopedics, Zhengzhou Central Hospital Affiliated to Zhengzhou University, Zhengzhou, China; ^3^ Center of Stem Cell and Regenerative Medicine, Zhengzhou Central Hospital Affiliated to Zhengzhou University, Zhengzhou, China

**Keywords:** proton pump inhibitor, osteoporosis, network toxicology, molecular docking, molecular dynamics simulation

## Abstract

**Background:**

Proton pump inhibitors (PPIs) are widely used for the treatment of acid-related disorders, but long-term use has been increasingly associated with an elevated risk of osteoporosis. However, the underlying molecular mechanisms and specific targets of PPIs-induced bone loss remain poorly understood. This study aimed to explore the molecular mechanisms and key genes of PPIs-induced osteoporosis using network toxicology, molecular docking, and molecular dynamics simulations.

**Methods:**

We identified common targets of four widely used PPIs (omeprazole, lansoprazole, pantoprazole, and rabeprazole) and osteoporosis by screening large-scale biological databases. A protein-protein interaction network was constructed, and key hub genes were determined based on topological parameters such as degree, betweenness centrality, and closeness centrality. Enrichment analysis was performed to explore the biological processes, cellular components, molecular functions, and KEGG pathways associated with the overlapping targets. Molecular docking was conducted to evaluate the binding affinities between PPIs and their potential targets, and molecular dynamics simulations were employed to assess the stability of these interactions over time.

**Results:**

We identified 35 potential targets for omeprazole-induced osteoporosis, 39 for lansoprazole, 29 for pantoprazole, and 29 for rabeprazole. Topological analysis of the protein-protein interaction networks revealed the hub genes for each PPI: epidermal growth factor receptor (EGFR) for omeprazole, estrogen receptor 1 (ESR1) for lansoprazole, EGFR for pantoprazole, and Proto-oncogene tyrosine-protein kinase SRC for rabeprazole. Molecular docking demonstrated strong and stable binding affinities between PPIs and their respective targets, with binding energies all below −5 kcal/mol. Molecular dynamics simulations confirmed the structural stability of these complexes, characterized by low root mean square deviation and root mean square fluctuation values and consistent hydrogen bond formation.

**Conclusion:**

This study identified EGFR, ESR1, and SRC as key regulatory genes in PPIs-induced osteoporosis, highlighting their roles in bone metabolism. The stable interactions between PPIs and these targets suggest their involvement in bone loss, providing a foundation for future experimental validation.

## 1 Introduction

Osteoporosis is a systemic bone disease characterized by reduced bone density and deterioration of bone microarchitecture, posing a significant global public health challenge (Subarajan et al., 2024). While traditional perspectives primarily attribute osteoporosis to aging, hormonal imbalances, and nutritional deficiencies, drug-induced secondary osteoporosis has garnered increasing attention in recent years (Wang et al., 2023). Notably, multiple studies have established a positive correlation between prolonged use of proton pump inhibitors (PPIs)—one of the most widely prescribed gastric acid inhibitors worldwide—and an elevated risk of osteoporosis (Targownik et al., 2008; Fattahi et al., 2019; Bioletto et al., 2024).

Since the introduction of omeprazole in 1988, PPIs have revolutionized the management of gastroesophageal reflux disease, peptic ulcers, and related conditions by irreversibly inhibiting the H^+^/K^+^-ATPase in gastric parietal cells (Strand et al., 2017). A systematic review of global trends and practices revealed that approximately 25% of adults have a history of long-term PPIs use, with one-quarter of these patients taking the medication for more than 1 year (Shanika et al., 2023). However, as the population using PPIs continues to expand, concerns regarding their long-term adverse effects on skeletal health have become increasingly prominent (Haastrup et al., 2018). Large-scale cohort studies have shown that long-term use of PPIs is associated with a 30%–50% increased risk of hip fractures, with this association demonstrating both dose- and time-dependency (Yang et al., 2006; Targownik et al., 2008). Notably, most existing studies attribute PPIs-induced bone metabolic disorders to the traditional hypothesis that gastric acid suppression leads to impaired calcium absorption (Wright et al., 2008). However, clinical intervention trials have found that calcium supplementation alone fails to fully reverse the trend of declining bone mineral density in PPIs users, suggesting the presence of deeper mechanisms independent of calcium homeostasis (Zhao et al., 2017).

**FIGURE 1:**
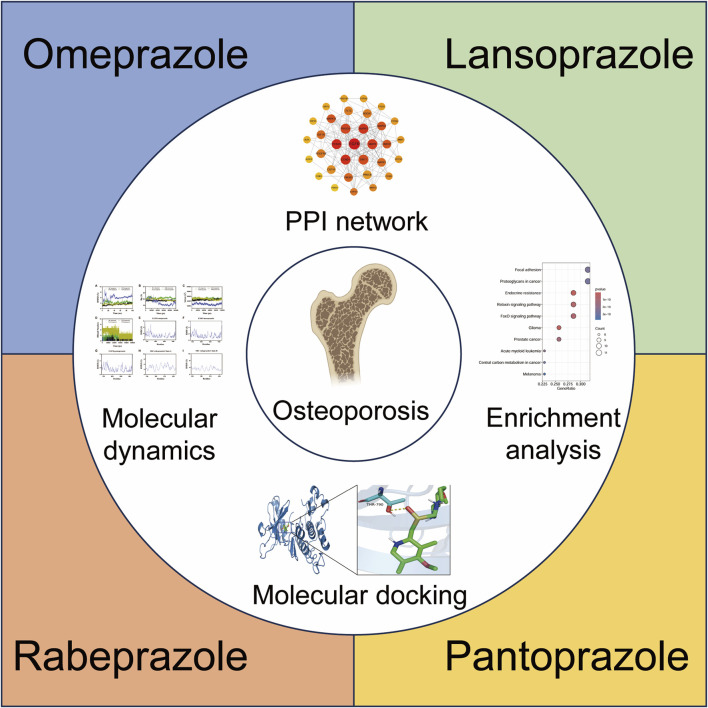
The schematic diagram of this study.

Network toxicology offers a powerful approach to overcoming the limitations of traditional research by constructing a multi-layered network of “compound-target-pathway-phenotype” interactions (Panagiotou and Taboureau, 2012). Molecular docking utilizes large-scale biological databases to efficiently screen the binding potential between PPIs and bone-related proteins (Pinzi and Rastelli, 2019). Furthermore, molecular dynamics simulation provides a dynamic assessment of the conformational changes within ligand-receptor complexes and allows for the quantitative evaluation of binding free energy and the contributions of key residues (Wu et al., 2022). In this study, we integrated network toxicology, molecular docking, and molecular dynamics simulation for the first time to systematically elucidate the molecular network through which PPIs interfere with bone metabolism (Figure 1). The potential targets identified in this study may offer valuable insights for clinical drug selection, structural optimization, and the balance between therapeutic efficacy and long-term safety.

## 2 Materials and methods

### 2.1 Target prediction of PPIs

In this study, the standard structures and canonical SMILES of four commonly used PPIs—omeprazole, lansoprazole, pantoprazole, and rabeprazole—were retrieved from the PubChem database (https://pubchem.ncbi.nlm.nih.gov/). Based on the retrieved data, target prediction was performed using the STITCH (http://stitch.embl.de/) and SwissTargetPrediction (http://www.swisstargetprediction.ch/) databases. The keywords “omeprazole”, “lansoprazole”, “pantoprazole”, and “rabeprazole” were used, with the species restricted to “*Homo sapiens*” to identify potential human-specific targets. The predicted target names were standardized using the UniProt database (https://www.uniprot.org/), and duplicate entries were removed to ensure data consistency and accuracy.

### 2.2 Screening of osteoporosis-related targets

Osteoporosis-related gene targets were identified through the integration of data from the GeneCards human gene database (https://www.genecards.org/). GeneCards is a searchable, comprehensive database that provides detailed and user-friendly information on all annotated and predicted human genes. This knowledge base automatically aggregates gene-centric data from approximately 200 web sources, including genomic, transcriptomic, proteomic, genetic, clinical, and functional datasets. The keyword “osteoporosis” was used for the search, and the screening threshold was set at a relevance score greater than the median value to exclude low-relevance genes.

### 2.3 Identification of potential targets for PPIs-induced osteoporosis and construction of the protein-protein interaction network

Venn diagrams were generated using the Venny 2.1 online tool to identify the overlapping targets between each PPIs and osteoporosis-related genes. The protein-protein interaction network was constructed using the STRING database (https://string-db.org/) with the minimum required interaction score set to a medium confidence level (0.4). Isolated targets without interactions were hidden from the network to improve clarity and focus on functionally relevant connections. The protein-protein interaction network was further analyzed and visualized using Cytoscape 3.8.2. In the network, the size and color of each node were ranked according to their degree value, where higher degree values indicated larger and darker-colored nodes, representing greater connectivity and potential biological significance.

### 2.4 Identification of hub genes in the protein-protein interaction network

Hub genes within the constructed protein-protein interaction network were identified through a comprehensive evaluation of three topological parameters: degree, betweenness centrality, and closeness centrality. The degree value represents the number of direct connections (edges) linked to a target node, indicating the extent of a protein’s direct interactions within the network. Betweenness centrality measures the proportion of the shortest paths passing through a given node relative to all shortest paths in the network, reflecting the node’s role as a central hub for information flow. Closeness centrality is defined as the inverse of the average shortest path length from a target node to all other nodes in the network, representing the efficiency of information transmission for that protein. All nodes in the network were ranked according to each of these metrics individually, and genes that consistently ranked highest across all three parameters were selected as hub genes. This approach enabled the identification of key regulatory nodes that play central roles in maintaining network connectivity and information flow.

### 2.5 Analysis of function and pathway enrichment for target genes

Gene Ontology (GO) functional enrichment analysis and Kyoto Encyclopedia of Genes and Genomes (KEGG) pathway enrichment analysis were performed using the “clusterProfiler” package in R 4.4.0. The “org.Hs.e.g.,.db” package was applied for gene ID conversion and standardization. Enriched terms with a significance threshold of *P* < 0.05 were considered statistically significant. Visualization of the enrichment results was conducted using the “enrichplot” and “ggplot2” packages.

### 2.6 Molecular docking

The two-dimensional (2D) structures of small-molecule ligands were obtained from the PubChem database (http://pubchem.ncbi.nlm.nih.gov/). These 2D structures were converted into three-dimensional (3D) structures using ChemOffice software and saved in the mol2 file format. For the protein receptors, crystal structures with high resolution were retrieved from the RCSB Protein Data Bank (http://www.rcsb.org/). Protein preparation, including the removal of water molecules, phosphate groups, and other heteroatoms, was performed using PyMOL software, and the cleaned structures were saved in the PDB file format. Molecular docking was carried out using AutoDock Vina 1.5.6 to investigate the interactions between the protein receptors and small-molecule ligands. Before docking, the protein and ligand structures were prepared using AutoDock Tools by adding hydrogen atoms, removing water molecules, and defining the torsional flexibility of the ligands. A docking grid box was set to encompass the predicted binding site, and the optimal ligand-receptor conformation was selected based on the docking score, with lower binding energy indicating higher binding affinity. Finally, the interactions between the ligand and key residues of the protein were visualized and analyzed using PyMOL and Discovery Studio 2019 software. Both 2D interaction diagrams and 3D structural analyses were performed to comprehensively illustrate the binding modes.

### 2.7 Molecular dynamics simulation

The protein-ligand complex was subjected to a 100 ns molecular dynamics simulation using Gromacs 2022. The protein was parameterized using the CHARMM 36 force field, and the ligand topology was constructed with the GAFF2 force field parameters. Periodic boundary conditions were applied, and the protein-ligand complex was placed in a cubic box. Water molecules were added using the TIP3P water model to fill the box. Electrostatic interactions were treated using the Particle Mesh Ewald (PME) method, and the Verlet algorithm was used for calculating non-bonded interactions. A 100,000-step equilibration was performed under the NVT (constant volume and temperature) ensemble followed by the NPT (constant pressure and temperature) ensemble, with a coupling constant of 0.1 ps and a simulation duration of 100 ps. Van der Waals and Coulomb interactions were calculated with a cutoff value of 1.0 nm. Finally, a 100 ns production molecular dynamics simulation was carried out at a constant temperature of 310 K and pressure of 1 bar using Gromacs 2022.

## 3 Results

### 3.1 Effects of omeprazole on osteoporosis

**FIGURE 2:**
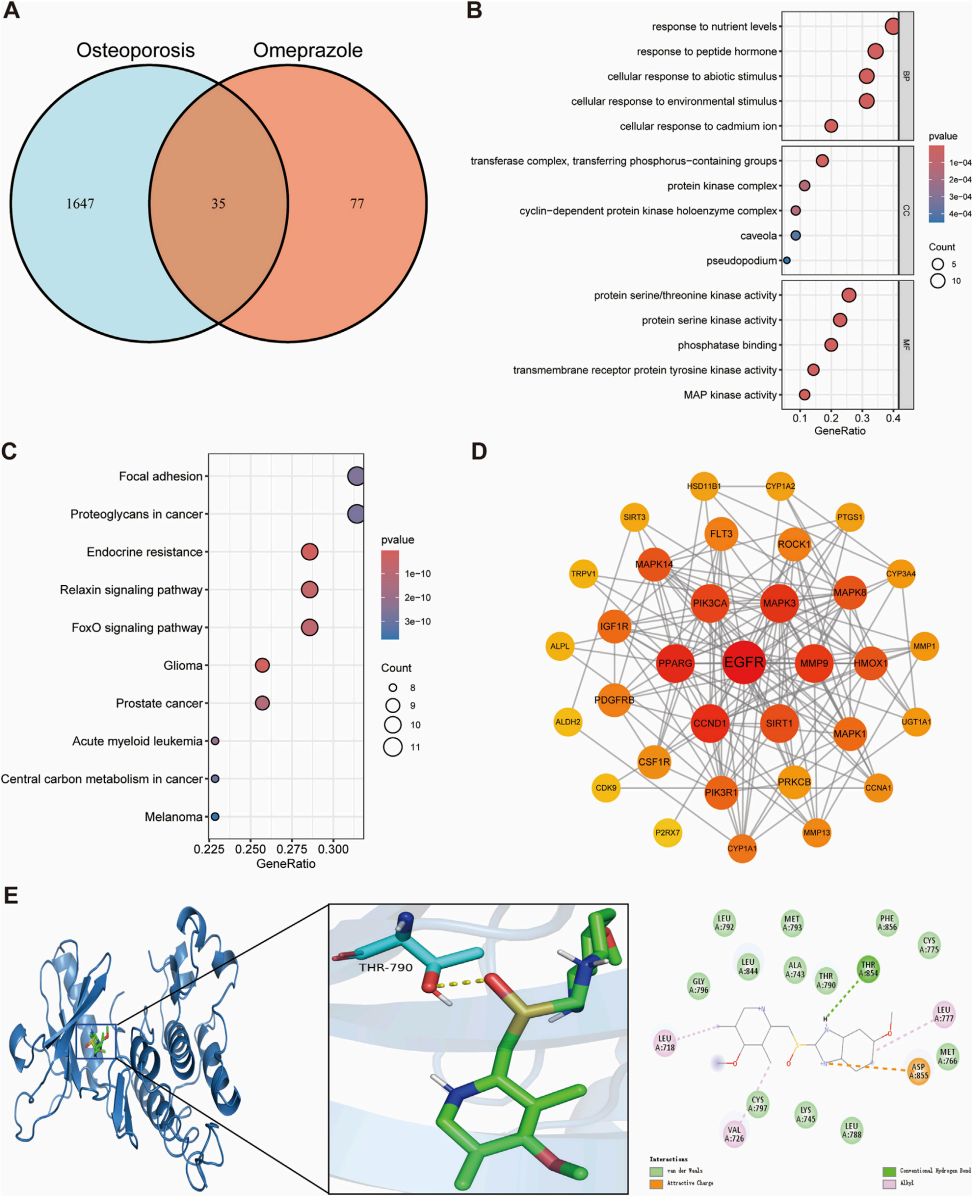
Network toxicology analysis and molecular docking of omeprazole-induced osteoporosis. **(A)** Identification of overlapping genes between omeprazole and osteoporosis. **(B)** GO functional enrichment analysis of overlapping genes. **(C)** KEGG pathway enrichment analysis of overlapping genes. **(D)** Protein-protein interaction networks of overlapping genes. **(E)** Molecular docking binding modes of omeprazole to EGFR.

In this study, we identified 35 potential targets of omeprazole associated with osteoporosis (Figure 2A). GO enrichment analysis revealed that the biological processes of these intersecting targets were primarily related to response to nutrient levels, response to peptide hormones, cellular response to abiotic stimuli, cellular response to environmental stimuli, and cellular response to cadmium ions (Figure 2B). Regarding cellular components, the targets were mainly associated with the transferase complex, protein kinase complex, and cyclin-dependent protein kinase holoenzyme complex (Figure 2B). In terms of molecular functions, the targets were predominantly involved in protein serine/threonine kinase activity and phosphatase binding (Figure 2B). KEGG pathway enrichment analysis indicated that the intersecting genes were significantly enriched in the focal adhesion, endocrine resistance, relaxin signaling pathway, and FoxO signaling pathway, suggesting their potential involvement in bone metabolism dysregulation induced by omeprazole (Figure 2C).

Through a comprehensive evaluation of degree, betweenness centrality, and closeness centrality, Epidermal growth factor receptor (EGFR) was identified as the hub gene with the highest topological importance in the protein-protein interaction network (Figure 2D; Supplementary Table S1). Molecular docking analysis demonstrated a binding energy of −9.2 kcal/mol between omeprazole and EGFR, indicating a strong and stable binding affinity. Detailed interaction analysis revealed that the THR854 residue of the EGFR receptor formed hydrogen bonds with omeprazole, while LEU718, VAL726, and LEU777 residues established hydrophobic interactions, further stabilizing the ligand-receptor complex (Figure 2E). These findings suggest that EGFR may play a crucial role in the mechanism underlying omeprazole-induced osteoporosis and highlight its potential as a key therapeutic target for future investigations.

### 3.2 Effects of lansoprazole on osteoporosis

**FIGURE 3:**
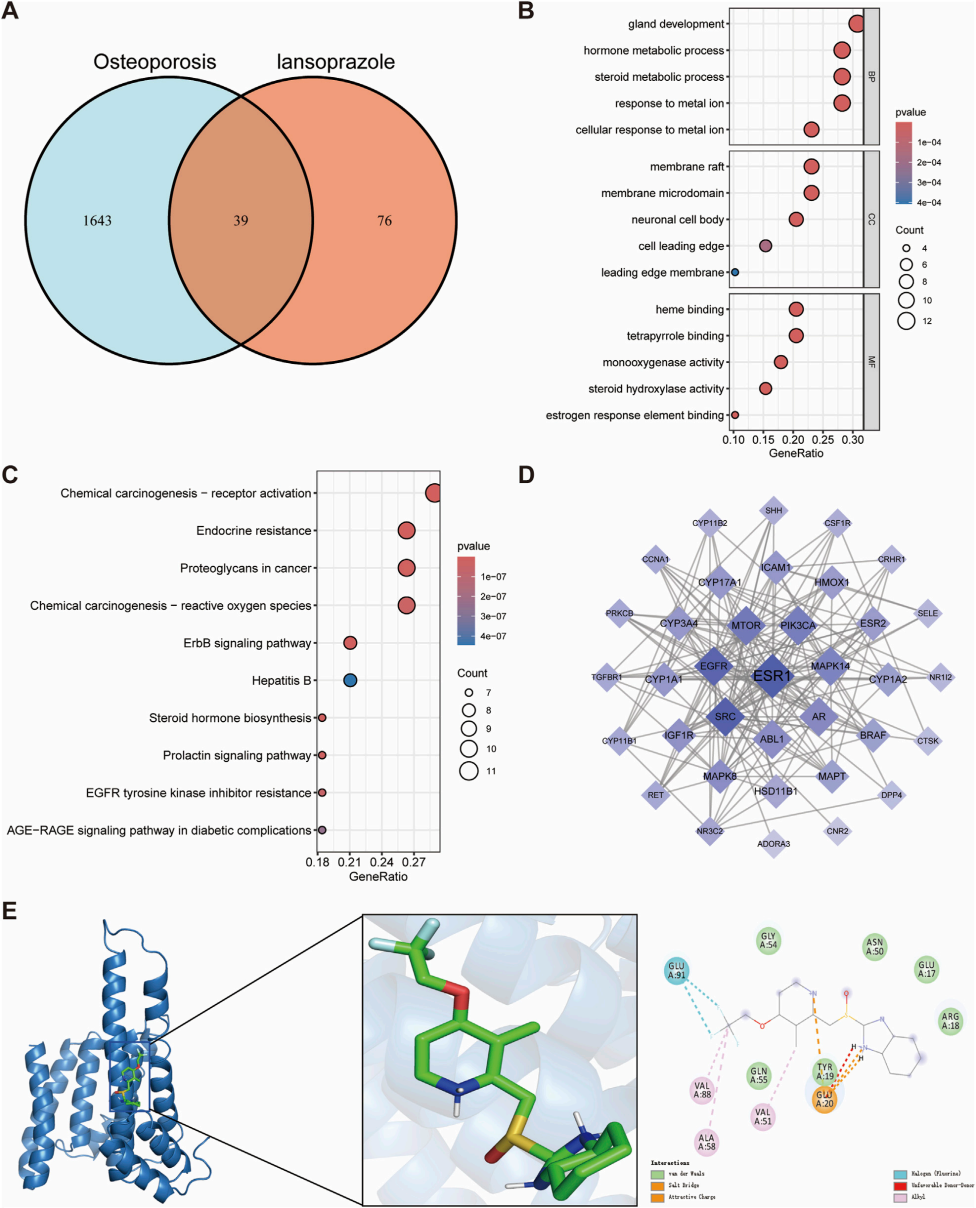
Network toxicology analysis and molecular docking of lansoprazole-induced osteoporosis. **(A)** Identification of overlapping genes between lansoprazole and osteoporosis. **(B)** GO functional enrichment analysis of overlapping genes. **(C)** KEGG pathway enrichment analysis of overlapping genes. **(D)** Protein-protein interaction networks of overlapping genes. **(E)** Molecular docking binding modes of lansoprazole to ESR1.

We next identified 39 potential targets of lansoprazole associated with osteoporosis (Figure 3A). GO enrichment analysis of the overlapping targets revealed that the key biological processes were primarily related to gland development, hormone metabolic processes, steroid metabolic processes, and response to metal ions (Figure 3B). In terms of cellular components, the targets were mainly enriched in the membrane raft, membrane microdomain, and neuronal cell body (Figure 3B). For molecular functions, the targets were predominantly involved in heme binding, tetrapyrrole binding, and monooxygenase activity (Figure 3B). KEGG pathway enrichment analysis indicated that the overlapping genes were significantly associated with the endocrine resistance, ErbB signaling pathway, and prolactin signaling pathway, suggesting their possible involvement in lansoprazole-induced disruption of bone metabolism (Figure 3C).

Estrogen receptor 1 (ESR1) was identified as the hub gene by evaluating the degree, betweenness centrality, and closeness centrality, highlighting its pivotal role in the lansoprazole-associated target network (Figure 3D; Supplementary Table S2). Molecular docking analysis showed a binding energy of −7.0 kcal/mol between lansoprazole and ESR1, indicating a moderate binding affinity. Interaction analysis revealed that the ALA58, VAL88, and VAL51 residues of the ESR1 receptor formed hydrophobic interactions with lansoprazole, contributing to the stability of the ligand-receptor complex (Figure 3E). These findings suggest that ESR1 may serve as a key regulator in the mechanism of lansoprazole-induced osteoporosis and provide insight into potential therapeutic targets for mitigating drug-induced bone loss.

### 3.3 Effects of pantoprazole on osteoporosis

**FIGURE 4:**
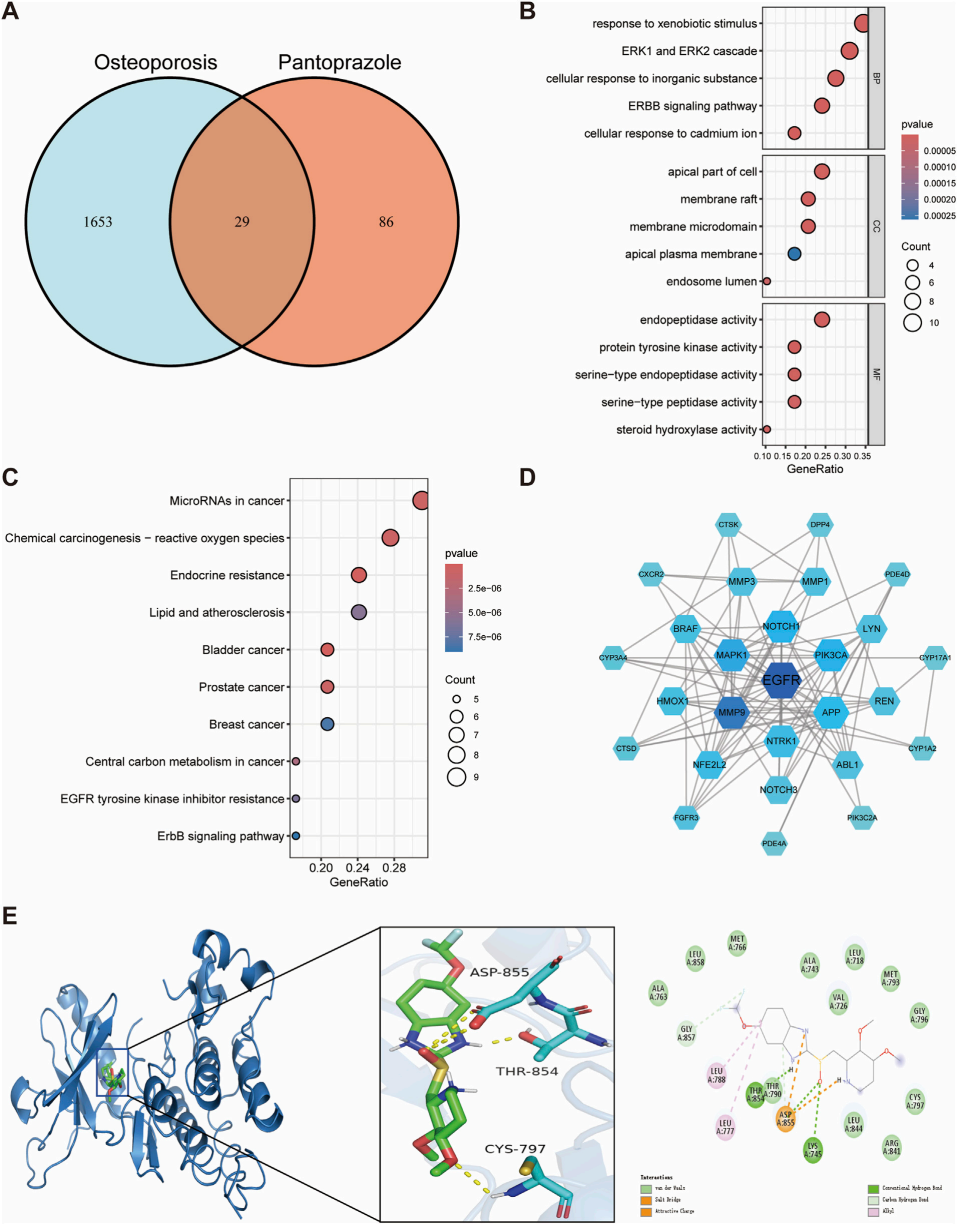
Network toxicology analysis and molecular docking of pantoprazole-induced osteoporosis. **(A)** Identification of overlapping genes between pantoprazole and osteoporosis. **(B)** GO functional enrichment analysis of overlapping genes. **(C)** KEGG pathway enrichment analysis of overlapping genes. **(D)** Protein-protein interaction networks of overlapping genes. **(E)** Molecular docking binding modes of pantoprazole to EGFR.

In this study, 29 potential targets of pantoprazole related to osteoporosis were identified (Figure 4A). GO enrichment analysis showed that the overlapping targets were mainly involved in response to xenobiotic stimulus, ERK1 and ERK2 cascade, cellular response to inorganic substances, ERBB signaling pathway, and cellular response to cadmium ions (Figure 4B). Regarding cellular components, these targets were predominantly associated with the apical part of the cell, membrane raft, and membrane microdomain (Figure 4B). In terms of molecular functions, the targets were enriched in endopeptidase activity, protein tyrosine kinase activity, and serine-type endopeptidase activity (Figure 4B). KEGG pathway enrichment analysis indicated that the intersecting genes were primarily involved in endocrine resistance, EGFR tyrosine kinase inhibitor resistance, and the ErbB signaling pathway, underscoring their potential role in pantoprazole-induced disturbances in bone metabolism (Figure 4C).

The EGFR was again identified as the hub gene through a comprehensive assessment of degree, betweenness centrality, and closeness centrality, suggesting its critical position in the pantoprazole-associated protein interaction network (Figure 4D; Supplementary Table S3). Molecular docking analysis revealed a binding energy of −8.6 kcal/mol between pantoprazole and EGFR, indicating a strong binding affinity. Further interaction analysis showed that the THR854 and LYS745 residues of the EGFR receptor formed hydrogen bonds with pantoprazole, while LEU788 and LEU777 residues established hydrophobic interactions, contributing to the stability of the ligand-receptor complex (Figure 4E). These findings reinforce the potential involvement of EGFR in pantoprazole-induced osteoporosis and highlight its importance as a therapeutic target for addressing bone metabolism disorders associated with long-term PPIs use.

### 3.4 Effects of rabeprazole on osteoporosis

**FIGURE 5:**
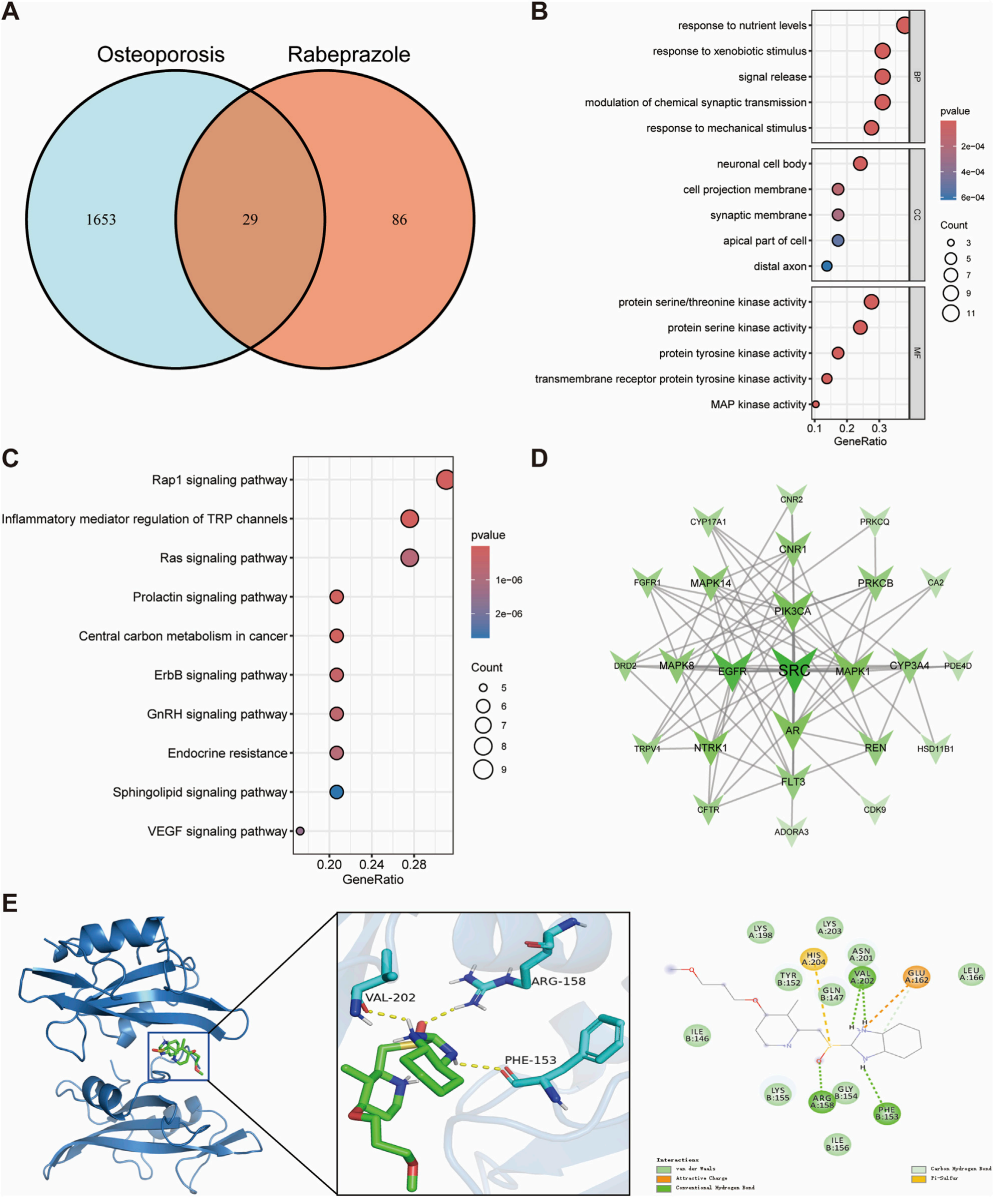
Network toxicology analysis and molecular docking of rabeprazole-induced osteoporosis. **(A)** Identification of overlapping genes between rabeprazole and osteoporosis. **(B)** GO functional enrichment analysis of overlapping genes. **(C)** KEGG pathway enrichment analysis of overlapping genes. **(D)** Protein-protein interaction networks of overlapping genes. **(E)** Molecular docking binding modes of rabeprazole to SRC.

We also identified 29 potential targets associated with the effects of rabeprazole on osteoporosis (Figure 5A). GO enrichment analysis demonstrated that the biological processes of these targets were predominantly involved in response to nutrient levels, response to xenobiotic stimulus, signal release, and response to mechanical stimulus (Figure 5B). For cellular components, the targets were mainly enriched in the neuronal cell body, cell projection membrane, and synaptic membrane, indicating their relevance to cell signaling and structural integrity (Figure 5B). The molecular functions were closely related to protein serine/threonine kinase activity, transmembrane receptor protein tyrosine kinase activity, and MAP kinase activity (Figure 5B). KEGG pathway enrichment analysis revealed that the potential targets were associated with pathways such as the Rap1 signaling pathway, inflammatory mediator regulation of TRP channels, Ras signaling pathway, and prolactin signaling pathway (Figure 5C).

Proto-oncogene tyrosine-protein kinase SRC was identified as the hub gene through an integrated evaluation of degree, betweenness centrality, and closeness centrality, underscoring its central role in the protein interaction network influenced by rabeprazole (Figure 5D; Supplementary Table S4). Molecular docking analysis indicated a binding energy of −5.9 kcal/mol between rabeprazole and SRC, suggesting a moderate binding affinity. Detailed interaction analysis revealed that ARG158, PHE153, and VAL202 residues of the SRC receptor formed hydrogen bonds with rabeprazole, contributing to the stability and specificity of the ligand-receptor complex (Figure 5E). These results suggest that SRC plays a pivotal role in the molecular mechanisms through which rabeprazole may disrupt bone metabolism, offering a promising target for further investigation into the long-term skeletal effects of proton pump inhibitors.

### 3.5 Molecular dynamics simulation for PPIs and hub targets

**FIGURE 6:**
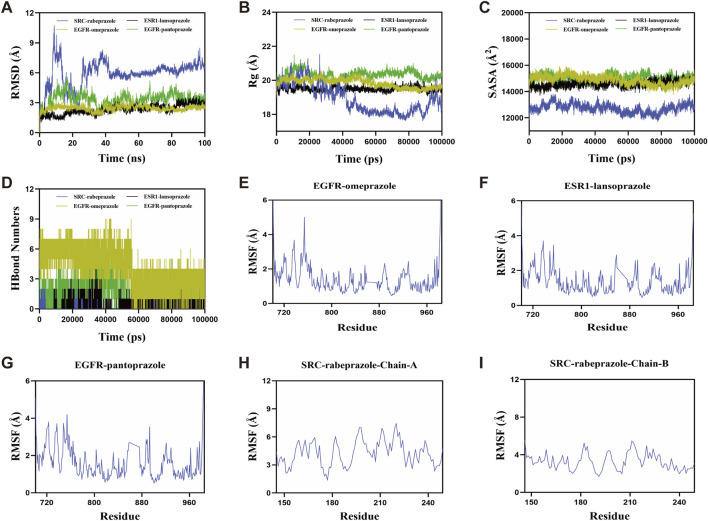
Molecular dynamics simulations of PPIs with target proteins. **(A)** Root mean square deviation. **(B)** Radius of gyration. **(C)** Solvent-accessible surface area. **(D)** The number of hydrogen bonds. **(E–I)** Root mean square fluctuation.

Molecular dynamics simulation was conducted to evaluate the binding stability between PPIs and their respective target proteins. The root mean square deviation (RMSD) was used as a key indicator of the conformational stability of the protein-ligand complexes, reflecting the deviation of atomic positions from their initial states. Lower RMSD values indicate greater structural stability. As shown in Figure 6A, the EGFR-omeprazole complex reached equilibrium after 60 ns, with RMSD fluctuations stabilizing around 2.6 Å. The ESR1-lansoprazole complex achieved equilibrium after 40 ns, fluctuating around 3 Å. The EGFR-pantoprazole complex stabilized at 20 ns, with an RMSD around 3.7 Å, while the SRC-rabeprazole complex reached equilibrium after 45 ns, with larger RMSD fluctuations around 6.7 Å. These results indicate that all four PPIs-target protein complexes exhibit relatively high binding stability. Further analysis of radius of gyration (Rg) and solvent-accessible surface area (SASA) revealed slight fluctuations during the simulation, indicating minor conformational changes in the EGFR-omeprazole, ESR1-lansoprazole, EGFR-pantoprazole, and SRC-rabeprazole complexes (Figures 6B,C).

Hydrogen bonds (H-bonds) play a crucial role in stabilizing protein-ligand interactions. As shown in Figure 6D, the number of hydrogen bonds varied over the simulation time for each complex. The EGFR-omeprazole complex maintained 0–9 hydrogen bonds, with an average of seven hydrogen bonds most frequently observed. The ESR1-lansoprazole complex showed 0–5 hydrogen bonds, averaging around two hydrogen bonds. The EGFR-pantoprazole complex displayed 0–7 hydrogen bonds, with an average of three hydrogen bonds, while the SRC-rabeprazole complex exhibited 0–2 hydrogen bonds, most commonly maintaining one hydrogen bond. These results demonstrate strong hydrogen bonding interactions, contributing to the stability of the complexes. The root mean square fluctuation (RMSF) was used to assess the flexibility of amino acid residues within the protein complexes. Lower RMSF values indicate greater structural rigidity and higher stability. Across the EGFR-omeprazole, ESR1-lansoprazole, EGFR-pantoprazole, and SRC-rabeprazole complexes, RMSF values remained relatively low, mostly below 4 Å, suggesting limited residue flexibility and enhanced stability (Figures 6E–I).

In summary, the EGFR-omeprazole, ESR1-lansoprazole, EGFR-pantoprazole, and SRC-rabeprazole complexes exhibited stable binding, strong hydrogen bonding interactions, and low conformational flexibility. These findings indicate favorable binding interactions between PPIs and their respective target proteins, supporting their potential roles in modulating bone metabolism.

## 4 Discussion

This study systematically explored the potential molecular mechanisms and targets of PPIs-induced osteoporosis by integrating network toxicology, molecular docking, and molecular dynamics simulations. Through extensive screening of biological databases and the construction of a protein-protein interaction network, we successfully identified the potential targets associated with osteoporosis for four commonly used PPIs—omeprazole, lansoprazole, pantoprazole, and rabeprazole. Furthermore, by evaluating topological parameters, we identified EGFR, ESR1, and SRC as key regulatory genes. These findings provide a novel perspective for understanding the molecular basis by which PPIs may contribute to the development of osteoporosis.

Although the molecular functions of the potential targets associated with omeprazole, lansoprazole, pantoprazole, and rabeprazole in osteoporosis exhibit heterogeneity, these targets are significantly enriched in the endocrine resistance and ErbB signaling pathways. This finding suggests that PPIs may induce osteoporosis by disrupting key cross-talk nodes within these pathways, thereby reshaping the balance of hormonal responses and growth factor signaling in the bone microenvironment. The association between endocrine resistance and osteoporosis is well-documented. Bone is an active endocrine organ, and its remodeling in adulthood depends on the balance between hormones such as estrogen and insulin. On one hand, insulin resistance reduces the activity of osteoblasts, leading to decreased bone matrix synthesis and mineralization; on the other hand, insulin resistance promotes osteoclastogenesis and enhances osteoclast activity, accelerating bone resorption (Greere et al., 2023). In postmenopausal women, the insufficiency of sex steroids, combined with age-related factors, further contributes to the development of insulin resistance (De Paoli et al., 2021). The ErbB signaling pathway’s involvement in bone metabolism has also been recognized. ErbB receptors, particularly ErbB3, interact with proteins like sclerostin to regulate osteoblast activity and bone formation (Lin et al., 2008). Disruptions in ErbB signaling can impair bone remodeling processes, potentially contributing to osteoporosis development.

The identification of EGFR, ESR1, and SRC as key regulatory hub genes provides valuable insight into the potential molecular mechanisms underlying PPIs-induced osteoporosis. EGFR is a well-established regulator of cell proliferation, differentiation, and survival, and its role in bone metabolism has gained increasing attention. EGFR signaling activation not only promotes osteoblast differentiation but also enhances the proliferation and survival of bone progenitor cells by upregulating early growth response two expression (Chandra et al., 2013; Linder et al., 2018). Conversely, the downregulation of EGFR signaling plays a physiological role during aging by reducing enhancer of zeste homolog two expression, which leads to the senescence of bone progenitor cells and a decline in bone formation on the endosteal surface of cortical bone (Liu et al., 2019). The disruption of EGFR signaling by PPIs, as suggested by the strong binding affinity observed in molecular docking and the stability seen in molecular dynamics simulations, could lead to an imbalance between bone formation and resorption, contributing to bone loss and increased fracture risk.

ESR1 is a key mediator of estrogen’s protective effects on the skeletal system. Estrogen signaling through ESR1 inhibits osteoclastogenesis and bone resorption while promoting osteoblast survival and bone formation (Khalid and Krum, 2016). The decline in estrogen levels, particularly in postmenopausal women, is a major risk factor for osteoporosis, and alterations in ESR1 expression or function can exacerbate this effect (Bajpai et al., 2024; Yao et al., 2025). Our findings suggest that lansoprazole has a strong interaction with ESR1, potentially interfering with estrogen signaling. This disruption could impair the balance between bone resorption and formation, further increasing the risk of osteoporosis, especially in populations already vulnerable to estrogen deficiency.

SRC is a critical regulator of bone metabolism. In SRC-deficient osteoclasts, the formation of the sealing zone and ruffled border is impaired, leading to compromised bone resorption activity (Boyce et al., 1992). In contrast to the loss of osteoclastic function, SRC-deficient mice exhibit an increase in osteoblast numbers and accelerated bone formation (Marzia et al., 2000). Moreover, studies in mouse models have demonstrated that the SRC inhibitor dasatinib enhances bone mass by simultaneously inhibiting bone resorption and promoting bone anabolism (Garcia-Gomez et al., 2012). Consistent with these findings, our molecular docking and dynamics analysis revealed a stable interaction between rabeprazole and SRC, suggesting that rabeprazole may influence SRC activity, thereby contributing to the development of osteoporosis.

Unlike previous studies that mainly focused on clinical observations, our study used a multilevel approach to reveal the complex molecular interactions underlying PPIs-induced bone loss. The integration of large-scale biological databases and rigorous topological analysis improved the reliability and reproducibility of our findings. Furthermore, molecular docking and molecular dynamics simulations confirmed the stable interactions between PPIs and their target proteins, providing strong support for the mechanistic hypothesis proposed in this study. However, this study still has some limitations. First, the predictions and findings are based on bioinformatics analysis and require further experimental validation. The interactions between PPIs and the identified hub genes (EGFR, ESR1, and SRC) should be confirmed through *in vitro* and *in vivo* studies to establish their biological significance. Second, the study does not fully consider the potential off-target effects of PPIs on other organs or systems, which may contribute to osteoporosis through indirect mechanisms. Third, although we identified key signaling pathways such as endocrine resistance and ErbB signaling, interpretation of these enrichment results should be approached with caution. Many genes possess pleiotropic functions and are involved in multiple biological processes, leading to the statistical enrichment of pathways—such as those related to cancers—that may not be directly relevant to bone metabolism. These outcomes reflect the intrinsic limitations of enrichment-based approaches, including potential biases from pathway database annotations and the risk of identifying biologically non-specific associations. Therefore, we focused our interpretation on pathways with clearer connections to bone health. Finally, the study focuses on only four widely used PPIs, and the effects of newer or less commonly used PPIs on bone metabolism need to be investigated for a more comprehensive understanding.

## 5 Conclusion

This study systematically explored the potential molecular mechanisms and targets of PPIs-induced osteoporosis through an integrated approach combining network toxicology, molecular docking, and molecular dynamics simulations. We identified EGFR, ESR1, and SRC as key regulatory hub genes, suggesting their crucial roles in bone metabolism and the development of osteoporosis. These findings were strengthened by the stability of PPIs-target protein interactions demonstrated through molecular docking and molecular dynamics simulations. This study not only provides novel insights into the molecular basis of PPIs-induced bone loss but also highlights potential targets for future therapeutic interventions. Further experimental validation and clinical studies are needed to confirm these results and explore their broader implications in osteoporosis management.

## Data Availability

The original contributions presented in the study are included in the article/Supplementary Material, further inquiries can be directed to the corresponding author.
